# Game over: dissecting the overlooked health harms of modern sports

**DOI:** 10.3389/fspor.2026.1753432

**Published:** 2026-02-18

**Authors:** Bilal Irfan, Kaden Venugopal, Ali Rehman, Rayyan Latif, Abdallah Abu Shammala, Roberto Daniel Sirvent

**Affiliations:** 1 Center for Bioethics, Harvard Medical School, Boston, MA, United States; 2 Center for Surgery and Public Health, Brigham & Women’s Hospital, Boston, MA, United States; 3 Department of Neurology, University of Michigan Medical School, Ann Arbor, MI, United States; 4 Department of Epidemiology, University of Michigan School of Public Health, Ann Arbor, MI, United States; 5 Faculty of Health Sciences, University of Ottawa, Ottawa, ON, Canada; 6 Wayne State University, Detroit, MI, United States; 7 Michigan State University College of Osteopathic Medicine, East Lansing, MI, United States; 8 Faculty of Medicine, Islamic University of Gaza, Gaza City, Palestine; 9 European Gaza Hospital, Khan Yunis, Palestine; 10 Department of Global Health and Social Medicine, Harvard Medical School, Boston, MA, United States

**Keywords:** physical activity, sport and labor, sport and militarism, sport-health ideology, sports economy

## Abstract

This article interrogates the “sport-health ideology” that equates organized sport with population wellbeing, arguing that it conflates the proven benefits of routine physical activity with the distinct injury profiles, labor relations, and political functions of modern sport. Synthesizing scholarship from sociology, public health, and bioethics, we show how elite and commercialized sport institutionalizes a culture of risk linked to musculoskeletal trauma, neurodegeneration, and normalized pain; embeds corporate sponsorships that promote unhealthy consumption; and operates through biopolitical regimes that intensify bodily manipulation for performance while denying comparable care to non-athlete publics. We trace the sport economy's dependence on precarious and exploitative labor, from collegiate exploitation to mega-event construction, and examine how patriotic pageantry and media partnerships constitute a “sport-security nexus” that aestheticizes U.S. militarism and its downstream health harms, including in Iraq and Gaza. We argue that sport bioethics must move beyond doping and concussion to treat labor rights, occupational and environmental standards, sponsorship governance, and demilitarization as first-order determinants of health. Redirecting investment toward low-risk, community-based movement, enforcing transparent safety surveillance across athletes and event workers, and severing ties with harmful industries are necessary to align sport with health justice.

## Introduction

Organized sports can often capture public attention with images of sculpted bodies in perpetual motion, marketing an alluring promise that competitive play is not merely thrilling entertainment, but a direct conduit to collective wellbeing. Triathletes, footballers, gymnasts, urban runners, and other elite athletes are routinely celebrated as paragons of vitality, their exertions mobilized in public-health campaigns that urge citizens to “get active” for the sake of their hearts, minds, and even national economies. Yet beneath the veneer of televised heroism lies a more complicated story in which pain, premature morbidity, and large-scale social harms are neither accidental nor peripheral but often woven into the very fabric of contemporary athletics. This analysis begins with this paradox.

We contend that the dominant “sport-health ideology,” the notion that sport is inherently, automatically health-promoting, can obscure a range of biophysical injuries and sociopolitical harms that are too often sidelined in both medical discourse and sociological debate. By interrogating how commercial sponsorship, labor exploitation, and militarized spectacle are normalized within sporting practice, we argue that academic engagement with competitive sport and health can reach far beyond the conventional focus on physical activity, cardiovascular benefit, or even the now-familiar concerns about concussion.

A departure point of ours is the influential critique advanced by Ivan Waddington and Andy Smith, who assert that evidence lauding the salutary effects of physical activity, exercise, and play is routinely transposed onto competitive sport without attending to the specific organisational pressures and injury profiles that distinguish the two (1). Their research shows that while moderate, rhythmic exercise can mitigate the burdens of non-communicable disease, elite sport, as well as its recreational counterparts, can assume a culture of risk, sustained by economic imperatives and masculinity codes that encourage athletes to “play hurt” and valorize violence on the field. The resulting pattern of negative health impacts is concerning: musculoskeletal trauma, osteoarthritis, and emerging neurodegenerative conditions cluster around sporting populations at rates seldom acknowledged in health promotion leaflets (2). Dominic Malcolm's synthesis further advances this critique, detailing how corporate sponsorship of sporting events by alcohol, fast-food, and sugar-sweetened beverage industries can entwine sport with consumptive practices that directly contradict public-health advice (3). Furthermore, the normalisation of sports betting through ubiquitous in-broadcast advertisements and team/league partnerships with wagering firms as a routine part and extension of the experience of consuming sports media can amplify health risks associated with gambling. Together, these insights provide the conceptual scaffolding for our first claim: the sport-health ideology persists in part by conflating qualitatively disparate activities (competitive organized sport and physical activity) under a single benevolent rubric (“health”) and by disavowing sport's unintended consequences.

The potential damaging health impacts of sport are not limited to those who wear the jersey. Parissa Safai's analysis of what constitutes medicalised and scientised bodies reveals how contemporary athleticism can depend on a biopolitical regime in which corporeal limits are hammered, welded, and chemically modulated in pursuit of performance (4). Simply put, elite athletes are often subjected to bodily manipulations to boost performance, promoting political systems that value economic interest over personal wellbeing. Athletes' bodies can thus become experimental sites for novel therapies and enhancement technologies, yet the same political economy that funds platelet-rich plasma injections for athletes may tolerate chronic underfunding of community clinics in the post-industrial neighbourhoods whose residents fill stadiums and consume branded merchandise. Similarly, the insurance companies who promote and provide these therapies for professional athletes at times restrict their use within elderly populations who may be at a greater benefit from their use. For instance, joint pain and inflammation from osteoarthritis, commonly found in elders, is a frequent illness that sports medicine physicians treat (5). Non-surgical standard of care for OA includes pharmacological and non-pharmacological treatments for pain management and functioning, such as physiotherapy, nonsteroidal anti-inflammatory drugs (NSAIDs), and joint injections (e.g., corticosteroids) (6). If the patient's condition does not improve with these treatments, other therapies can include Monovisc (hyaluronic acid) and platelet-rich plasma injections (7). In Ontario, Canada's most populous province, the Ontario Health Insurance Plan (OHIP) does not cover either therapy, given their high costs or perceived lack of medical necessity (8, 9). While professional athletes often have extensive insurance coverage and access to out-of-pocket payment options through their organizations, the many patients in need of these therapies do not (10). As these therapies can cost thousands of dollars, patients without financial means for these therapies can be left with sustained suffering (11). Safai's ethnographic vignettes of Toronto's gentrifying fitness factories also does show somewhat how sport simultaneously advertises individualised lifestyle solutions and distracts attention from structural determinants of health such as job insecurity, racialized policing, and environmental degradation, all of which bear heavily on health. Her work, therefore, can widen the aperture of enquiry, prompting us to ask how the spectacle of what is seen as a form of elite fitness reflects and reproduces broader social inequities. Somewhat relatedly, scholars have described a sort of ablenationalism wherein there risks being a political fetishisation of hyper-able-bodiedness as being an ideal citizen (12). The sport-health ideology could inadvertently function as a cultural engine for this ideal by linking worth, discipline, and national virtue to a narrow image of what constitutes an athletic body.

Our critique largely targets elite, commercialised, and spectacle-driven sport systems, as well as their associated institutional environments that enable for the convergence of performance, media attention, and profit-driven calculations to convert bodies into assets to be used, injured, and discarded. It may be beyond the scope of this paper to examine whether all organised sport is intrinsically health-damaging, though there may be reasons to think so, yet some argue that many community-based, school, and youth sporting contexts can be health-promoting when they are non-exploitative, appropriately resourced, and oriented toward safe participation rather than commodified performance (13, 14). Nevertheless, many do indeed often critique competitive sport in itself, if even in a structural sense rather than blaming individual actors (13, 14).

Building on these foundational critiques, our analysis advances bioethical concerns against the sport-health narrative in three claims. We proceed from mapping the direct, individual bodily harms that are associated with sport participation to the indirect, collective health harms embedded in the sporting industry and its associated societal functions. In essence, we move from considering the actual playing of sports, to the sport economy, and then to the broader social and political infrastructures that organised sport often depend upon, help naturalise, and can reproduce. First, we illustrate how mega-events and professional leagues can rely on transnational labour regimes that externalise health harms onto marginalised workers. A vivid recent example is the hyper-visible stadium construction in Qatar, though comparable dynamics underpin collegiate sport in the United States and the continued invisibility of some of women's labour across media platforms (15, 16). Next, we examine the interface between competitive sport and imperial power, tracing how patriotic rituals, military fly-overs, and strategic partnerships can render American militarism palatable, even glamorous, at home while legitimizing health-damaging interventions abroad (17). The US National Football League's half-time tributes to the armed forces are not entirely benign pageantry. Rather, they are integral to what can be termed the sport-security nexus that normalizes war-related trauma and environmental toxicity in areas like Iraq and Gaza. Finally, we situate these critiques within bioethical discourse, arguing that prevailing debates, doping, concussion protocols, genetic engineering, may be necessary but currently insufficient. A genuinely comprehensive sport bioethics must also interrogate how sponsorship logics, labor hierarchies, and geopolitical alignments systematically shape the distribution of health benefits and harms.

Contours of these debates are already visible in the medical literature that wrestles with conflicts of interest among team doctors, the sociological studies that chart the gendered embodiment of pain, and in the public-health analyses that document rising obesity alongside competitive organized sport's global expansion (18). Yet the domains of literature seldom converse with each other, and even when they do, they rarely address how sport serves as a relay between local bodily experiences and global political economies. Therefore a central contribution of this paper is to stage a deliberate dialogue that draws and synthesizes insights from sociology of sport, bioethics, and public-health scholarship, fields that often diagnose adjacent problems (e.g., injury, harms of sponsorship, labour exploitation, militarised spectacle) without a shared explanatory frame that can link physical harms to the political economy. This analysis seeks to bridge that gap. Our aim is not to dismiss or demonize sport in its entirety, but rather to recalibrate analytic attention toward the conditions under which competitive sporting practices, if any, genuinely promote health and those under which it exacts unacceptable physiological or societal costs (13). In so doing, we hope to contribute to the growing realization within both the health sciences and sport studies that the moral and medical stakes of athletic culture extend well beyond sideline injury tents, reaching into factories, boardrooms, and conflict zones that are too often bracketed out of view.

## Separating the health consequences of physical activity and sports

Regular bodily movement, whether accumulated through brisk walking, household chores, or commuting by bicycle, constitutes one of the most powerful, low-cost determinants of human health documented to date. If viewed as constituting physical activity, it is a research proven behavior which can promote beneficial health in multiple domains such as cardiovascular, metabolic, cognitive, and emotional (19). Recent studies have shown that engaging in regular physical activity may significantly lower the risk of chronic conditions such as cardiovascular diseases, type 2 diabetes, and certain cancers (20–29). In a comprehensive 2024 systematic review and meta-analysis pooling 136 papers from 76 longitudinal and intervention studies and 2.6 million generally-healthy adults, Oja et al. found that recreational cycling cut coronary heart disease risk by 16% and all-cause, cancer, and cardiovascular mortality by 21%, 10%, and 20% respectively (30). Football can improve body composition, lipids, fasting glucose, blood pressure, resting cardiovascular function, cardiorespiratory fitness, and bone strength; handball can produce parallel gains in adiposity control and aerobic capacity; running may lower all-cause, cancer, and cardiovascular mortality by 23%, 20%, and 2%, while enhancing body composition and haemodynamic and aerobic indices; and swimming could reduce all-cause mortality by 24% alongside favourable shifts in adiposity and lipid profile. These together serve to demonstrate that the health dividends popularly attributed to sport are often overwhelmingly mediated by the moderate-to-vigorous physical activity (MVPA) embedded in these recreational modes, with evidence for other disciplines still too sparse for quantitative synthesis.

The benefits of physical activity span multiple physiological, psychological, and social domains: a 247-study mediation synthesis shows that regular movement can ease pain and fatigue while bolstering self-esteem, social connection, and resilience, mechanisms that could collectively translate into better mental health and overall well-being (31). What's more is that accelerometer dose-response work reveals that mortality risk could plummet once people move even modestly, roughly an extra hour of light activity or a few minutes of MVPA per day, an effect that persists across varied 24-hour movement patterns and emerges well below current guideline thresholds, serving to confirm that the decisive ingredient to good health is likely ordinary movement itself rather than organized competition (32, 33).

While organized sports are often associated with positive health outcomes, the data does point to the physical activity within sport rather than solely its competitive, institutional, or symbolic structures as the true health promoting factor. Physical engagement in activities such as running, swimming, and football can contribute to improved cardiometabolic profiles and lower mortality risk, yet these findings often stem from general populations engaged in low to moderate intensity forms of play rather than participants in commercialized or elite sport systems. These distinctions challenge the dominant sport-health ideology and the notion that sport equals good health: once physical activity becomes entangled with hyper-competitiveness, hierarchies, and constant evaluation, its health benefits can be compromised. A 2019 occupational-safety review of elite sport demonstrates that the prevailing win-at-all-costs ethos may reframe serious injuries as being part of routine workplace risks, can promote under-reporting and risk-taking (e.g., playing hurt), and ultimately may embed the celebration of pain as proof of commitment within high-performance sporting culture (34). Persistent structural faults, chief among them the commodification and exploitation of athletic labor, can also erode some lasting health gains that organized sport claims to deliver.

Organised sports often exist on a continuum of different settings which have meaningfully different profiles of risk and institutional incentives attached to them. On one hand there are community-based youth programmes and the likes, as well as school-related sporting activities in secondary education, which often can provide a form of social connection, structured routine, and be part of identity-building when coaching cultures are adequately prioritizing safety, inclusion, and development over a rigid focus on athletic outcomes. Then there are also competitive amateur and semi-professional environments, where often pressures to be selected, specialisation in specific areas of sport and role, and intensified burden of training could begin fermenting some of the ethos of normalizing injury. Beyond that one sees the market of elite and commercialised sports and their related systems, including media contracts, sponsorships, and career defining ventures, which could serve to reward certain forms of risk-taking. It is important to note that the risk, mode, and profile of injury is quite heterogeneous across different sports. For example, collision and high-contact sports tend to carry higher risks of sustaining acute trauma to the extremities or head, whereas many non-collision sports may have lower acute-trauma risk but could still generate a multitude of injuries, unhealthy training cultures, or cumulative health harms over time. Even if all sports do not confer the same sort of risks, the institutionalization of competition and performance in sports can at times convert otherwise healthy physical activity into a harm. The structure inherent in many sports settings may often not create the optimal environments for safety or inclusion, as the pressures of ranking, sorting, hierarchical, and evaluative aspects within sports can at times fundamentally affect one's psychological health and interpersonal relationships (e.g., conditional self-worth; your friendships might be affected because of competition and what use you are to your teammates, etc.). Furthermore, inclusion also often requires a contrasting exclusion (e.g., starting player vs. bench player; who gets cut; defeating the opponent is often a necessary part of competition - mutually exclusive goal attainment). There is also cause to consider whether identity building in terms of finding some kind of dignity or empowerment in sports is worth critiquing as there may be advancements in inclusion for otherwise unhealthy and/or violent activities that are framed as progress rather than seeking to challenge and dismantle harmful structures (35).

Consequently, public-health strategy could pivot toward forms of movement that are community-based, low-risk, and genuinely accessible, rather than solely those embedded in profit-driven sporting institutions. With that shift in view, we now turn to the global sport economy itself, where the same logics of extraction play out often across migrant worksites and supply chains (36).

## Global sporting promotes worker exploitation

In prevailing research on the social determinants of health, employment and working conditions have long been recognized as decisive loci of advantage and vulnerability. Contemporary occupational health scholarship describes employment as a sort of undervalued lever that could be used to improve population health (37). Yet discussions of the health impacts of labor seldom extend to the highly mediated and lucrative world of sport. The supposition that organized athletic competition is an intrinsically positive phenomenon has sometimes detracted researchers and policy-makers from the strenuous, frequently precarious, and at times lethal conditions endured by those who stage the spectacle. From the collegiate basketball star who generates millions for a university that withholds wages to the Bangladeshi migrant who erects a World-Cup stadium under desert heat, the modern sport industry mirrors, and often magnifies, the structural violence of late capitalism (38). In this section we aggregate evidence from recent scholarship to scrutinize how labour exploitation in sport is a consequential socio-political determinant of health. We situate this argument within traditions of health sociology and sport studies, provide illustrative cases of exploitation across different strata of the sport workforce, and then examine the 2022 FIFA World Cup in Qatar as paradigmatic of the commodification of athletic entertainment, the pursuit of geopolitical legitimacy, and the deterioration of worker health converged.

Capitalist sport and its labor markets can organize risk just as surely as they distribute income. Over the past three decades, high-income countries have observed a steep rise in non-standard, insecure, and digitally mediated forms of work that can concentrate hazardous exposures among people with the least bargaining power (37). Epidemiological syntheses confirm that sustained high-impact training and chronic overworking, irregular schedules, continuous job insecurity, and the absence of collective representation could predict worse cardiovascular, musculoskeletal, and mental-health outcomes (37). These risks to athletes are not entirely random, but somewhat patterned by gender, race, migration status, and class, thus creating a gradient of harm that could intensify with each point of social disadvantage. Within the athletic economy, the same logic is discernible, though frequently obscured by the glamour of elite performance. Elite sport is, in essence, could be seen as a form of ordinary work under extraordinary scrutiny: it may deserve the similar attention and analytic tools used to expose the deleterious effects of precarious employment in other sectors.

Several perspectives can be employed to clarify how and why sport labor can produce and exacerbate ill-health. Sociological approaches to medicine can emphasize the commodification of labor power, the extraction of surplus value, and the attendant degradation of working conditions manifest in heightened morbidity and mortality among workers (37). Occupational injuries do not end at the gate of the factory or the stadium–they could infiltrate households and communities through debt, disability, and truncated life chances, leading to social reproduction for future generations (39, 40). Within sport sociology, early analyses revealed the athletic field as a site where capitalist relations are both literal, where players sell their bodies to team managers, or a sort of owners, for the benefit of crowds, and symbolic, where ideologies of meritocracy are promoted and inequalities naturalized (41). More recent scholarship shows how global investment in clubs, leagues, and events functions as a sort of spectacle of accumulation, wherein states and corporations exchange capital for cultural prestige via organized sport, a social phenomenon now widely recognized as sportswashing (42–44).

Against this theoretical backdrop, concrete examples illuminate the varied modalities of exploitation in the sport labour process. In the United States, the NCAA's long-standing prohibition on salaries forced some collegiate athletes, including Black men in revenue-sports, to generate billions in media income while labouring without fair wages, social protection, or long-term health insurance (45). Although the 2021 judicial ruling on name, image, and likeness (NIL) rights enables some athletes to monetise personal brands in the U.S., the policy merely substitutes a gig-economy model of self-curated sponsorships for a living wage, thereby deepening stratification within teams and leaving most players unpaid (45). A somewhat parallel dynamic unfolds in women's professional sport, where marketing discourses celebrate empowerment yet conceal vast reservoirs of unpaid labour (46). Women football and softball players are often required to perform at community clinics, for social-media outreach, and conduct fan engagement tasks that are vital to league sustainability–sometimes without remuneration, rationalised as service to future generations (46, 47). In the realm of sports entertainment, an investigation into women's professional wrestling exposed a workplace riddled with catastrophic injuries, coerced sexualisation and sexual harassment by coaches/teammates, and psychological abuse, sometimes all under an employment classification that denies wrestlers occupational-health rights and collective bargaining (48–50). These cases are illustrative rather than exhaustive as they delineate a continuum of harms that stretches from the intermittent gig to the full-time but still precarious athlete-entertainer.

Among the most potent crystallisation of sport-related labour exploitation in recent memory is the construction and delivery of mega events, with the 2022 FIFA World Cup in Qatar representing an important case in point. Scholars of global sport governance observe that mega events have migrated toward authoritarian or semi-authoritarian hosts, a trend propelled by the strategic desires of states in the geopolitical periphery and the rent-seeking imperatives of Western-based sport federations (41–44). An assemblage model can be used to analyze the issue: capital-rich but prestige-poor states could purchase cultural legitimacy from prestige-rich but capital-seeking Western institutions. The result is a bidirectional process in which labour exploitation is not an unfortunate side-effect but a structural condition of the bargain (41). In Qatar the cost was measured in both currency, over USD 200 billion in infrastructural expenditure, and in the broken bodies of a largely South-Asian migrant workforce (51, 52). Investigative journalists and human-rights groups estimate that thousands of workers died in the twelve-year build-up to kick-off, victims of extreme heat, crushing workloads, wage theft, and a kafala sponsorship system that rendered them deportable for dissent (53–56). Although the Supreme Committee for Delivery & Legacy introduced voluntary welfare standards and touted modest reforms such as reimbursement of recruitment fees and the abolition of exit permits, these measures applied only to a sliver of the workforce and arrived after some of the most dangerous phases of stadium construction had ended (54, 55, 57). Furthermore, even the updated labour codes at times left workers without freedom of association, suppressing the foundational right to organise collectively for safer conditions (54).

Mega events also do carry the risk of generating de-localised environmental health burdens that are often actually omitted or neglected in prevailing narratives surrounding public health harms. For example, large-scale construction, coupled with an accelerated pathway to urban development and infrastructure projects, can serve to expand carbon emissions, material waste, water, and energy demands (58). They may also increase the risks of heat exposure and the local air quality, which can impact workers and low-income residents who live near such worksites or are displaced by them (58).

From a public-health perspective, the Qatar World Cup offers a stark example of the health inequities at play and the downstream health consequences that accrue. It demonstrates how the convergence of three forces, temporary demand for heavy labour, permissive transnational governance, and systemic racialised migration, can yield severe, predictable, yet politically tolerable health violations. International migrant workers frequently occupy a position diametrically opposed to the notion of an healthy immigrant effect, arriving with pre-existing vulnerabilities that are then amplified by exploitative labor conditions (37, 59, 60). The FIFA 2022 case confirms this observation: autopsies were rarely conducted, causes of death were recorded as stemming from natural causes despite the unambiguous risk of working in forty-degree celsius heat, and some families were left without compensation due to documentary lacunae (55). The attending rhetoric of the organisers exemplifies what is termed as a sort of tyranny of the table, the legal abstraction that can convert corporeal loss into pre-priced body parts, potentially severing injury from the moral universe that gives it meaning (39, 40). In this sense mega events do not merely reflect capitalist labour relations, they can actually intensify them under conditions of global visibility yet juridical invisibility.

Health consequences of such arrangements can extend beyond acute injury. Studies suggest correlations between precarious employment, migratory insecurity, and chronic occupational stress to elevated risks of cardiovascular disease, depression, substance abuse, and suicide (37, 61). Women professional wrestlers, for example, have been noted to have elevated opioid use for chronic pain management, and some have suffered from traumatic brain injury or even fatal overdoses relating to opioid use (48). For women athletes, the hidden labor of brand maintenance may contribute to anxiety, disordered eating, and social-media harassment, compounding the effects of absent/limited guranteed salaries and health coverage (45, 46). These outcomes are not necessarily accidental by nature, but rather may be expressions of the clear embodiment of exploitation (39). Capital accumulation in sport, whether routed through television contracts, sovereign wealth funds, or nostalgia-driven merchandising, can extract surplus by subordinating health to profit.

Nevertheless, exploitation is not entirely immutable. The migrant workers who struck South African stadium sites in 2010 under the idea of there being no world cup without attention to workers' rights did compel wage increases and catalysed international scrutiny, a precedent for the AFL-CIO and Building and Wood Workers’ International campaigns in Qatar (54, 62). College athletes in the United States, in leveraging public sympathy and legal challenges, were able to overturn the NCAA's NIL ban and expose the amateur myth while recasting players as workers with publicity rights (45). Women wrestlers, through collective storytelling and litigation, have documented the scale of abuse, prompting scrutiny of the World Wrestling Entertainment's monopolistic labour regime (48). Such movements serve to affirm the contention that health in the political world of sports is a terrain of struggle. When workers possess the organisational capacity to enforce safety standards, bargain for fair remuneration, and contest ideologies that naturalise their disposability, some of the effects of labour exploitation can be mitigated.

Perhaps sport can be seen as both a mirror and an amplifier of the broader labour market transformations. The shift toward precarious, gig-based, and migration-dependent employment, the intensification of digital surveillance, and the racialised distribution of occupational risk can find exaggerated expression within the multibillion-dollar sport economy. By tracing the health implications of these dynamics, from unpaid collegiate athletics to the cofferdams of Lusail, we showcase the necessity of integrating occupational-health expertise and empirical evidence with sport studies and medicine. Worker exploitation in sport is more than a mere peripheral curiosity in the domain of health; it is a central node in the global political economy of health. Future interventions could potentially benefit from transcending the celebratory rhetoric of empowerment that surrounds athletic performance and could confront the material conditions that sustain the spectacle. In recognizing athletes, stadium builders, and support staff as workers, worthy of the same rights and protections as laborers in other fields, competitive sport can be transformed from a site of embodied extraction into one of collective wellbeing.

## Sports, U.S. imperialism, and health

The modern U.S. empire is sustained through more than just the movement of fleets, diplomats, arms, and capital, but also through diffusing cultural currents that normalize conquest while masking its bodily consequences. Physicians working in Iraq, for example, still treat children whose blast injuries trace back to ordnance dropped during the 2003 U.S. military campaign; epidemiologists in Puerto Rico still chart excess mortality stemming from colonial fiscal austerity; psychiatrists on Pine Ridge still confront the psychological sequelae of land theft (63, 64). These disparate clinical vignettes point to what scholars have called the continued health components of imperialism, a patterned injury profile, trauma, malnutrition, infectious disease, toxic exposure, and despair that can flow predictably from foreign colonial rule and the violent extraction it requires (65, 66). What is often obscured is the cultural machinery that facilitates such injuries. Modern spectator sport is often a central gear in this machinery. The stadium, the television broadcast, and the socially sanctioned rituals that surround them can function as affective conduits, moving citizens from the quotidian to an emotional register in which martial violence appears inevitable, and even righteous. This section traces three nodes in that process: the stereotyping of Indigenous peoples through Native mascots; the post-2001 fusion of professional sport and the Pentagon; and the sporting establishment's mobilisation behind the current U.S-backed Israeli military assault on Gaza.

### Imperialism and the invention of modern sport

From its Victorian codification forward, modern sport has often travelled in the bellies of imperial vessels. Many Anglo-American administrators living in treaty ports, colonial garrisons, and naval bases used baseball, football, and track meets to drill subject populations in acquiescence and to declare Anglo physique as evolutionary ideals (67). Some Olympic revivalists explicitly linked athletic discipline to the notion of a civilizing mission, insisting that colonial subjects learn European games as a mark of fitness for limited self-government (68). In the United States, the project was simultaneously inward-facing: college football spectacles at the Carlisle Indian Industrial School were often staged as proof that Native youth could abandon supposed savagery for regulated Anglo aggression, thereby legitimizing the boarding-school system itself (69). These sporting laboratories were able to do more than just advertise empire: they produced a psychosocial template in which racial domination and bodily excellence appeared mutually reinforcing, an ideological legacy that continues to inform twenty-first-century U.S. foreign policy marketing (70).

### Native mascots: settler colonial imagery and indigenous health

The United States' imperial genealogy pre-dates 1898, as it arguably begins with the military-corporate occupation of the lands of hundreds of Indigenous nations (69, 71). Native mascots materialize that occupation in the affective space of sport. Some estimates suggest that at least 2,000 teams across the U.S. still employ native imagery, primarily through elementary, middle, and high school mascots, while professional franchises such as the Kansas City Chiefs continue to move millions of units of branded merchandise (72). A comprehensive review of empirical studies found that this imagery can carry measurable mental health risks for Indigenous youth, such as depressing self-esteem and intensifying anxiety during developmental years while identity formation is neurologically plastic (72). Field experiments are emblematic of some of these effects. One study found that after a brief exposure to the “Chief Wahoo” logo (the former logo of the Cleveland Guardians, previously Cleveland Indians, used until 2018), certain Indigenous high school students reported lower community worth and articulated fewer academic future possibilities than peers in control groups (72, 73). Indigenous students have also showed acute spikes in hostility and depressive affect after viewing ostensibly neutral Sioux imagery (72).

These harms are not merely psychological. The very existence of such mascots echoes harms to Indigenous peoples as foundational to U.S. sport, celebratory acts of discursive violence that facilitate material dispossession in the present (74). An analysis of congressional debates over the Washington Commanders (formerly known as the Washington Redskins until 2020) team name illustrates this feedback loop: lawmakers who defended the “Redskins” brand can often simultaneously advance legislation undermining tribal sovereignty and funding pipelines through treaty lands (73). The health sequelae are therefore bidirectional: mascots may wound Indigenous communities directly through stereotype threat and indirectly through the policies they legitimize, from chronic water insecurity on Navajo territory to the elevated suicide rates documented among Indigenous adolescents navigating racist school climates (72).

Even beyond just team names and logos, mega events can themselves actually serve to mobilize Indigenous culture as being a sort of spectacle through which colonial history is laundered into a narrative of country-wide harmony. Opening ceremonies at events do sometimes stage the Indigenous traditions and cultures as a sort of aesthetic heritage, which is being selectively celebrated while ignoring much of the ongoing land dispossession or structural inequities such communities facing (75). In a way, sports could act as a form of cultural pageantry that functions to offer symbolic recognition of specific cultures or traditions while leaving the material determinants of their health unspoken about.

### Patriotic pageantry and 21st-century U.S. adventurism

As mascots link settler colonialism with domestic fandom, post-9/11 pageantry ties U.S. global military campaigns to religious rituals. In the aftermath of 2001, the Department of Defense deepened its presence in professional sporting events by supplying color guards, bands, ceremonial flag-unfurling, and on-field enlistment spectacles, while recruiters staffed promotional booths and teams adopted military-style uniforms. In equating team performance with national service, audiences can develop a subliminal connection between winning a game and winning a war, reinforcing patriotic ideals and nationalist narratives. Together, these mutually beneficial arrangements turned televised sport competitions into powerful stages for patriotic theatre and recruitment drives (76). These productions helped transform sports arenas into vernacular recruiting depots while coercing dissenters into performative patriotism, a dynamic that renders refusal to stand during the seventh-inning rendition of “God Bless America” tantamount to treason (76). Implicit-association studies show that when college students who already harbour anti-Indigenous biases are exposed to Native-themed sport logos, they are more prone to stereotype a fictitious Native student as aggressive, an effect that does not occur with neutral or “White-themed” logos (72). Such exposure has been found to potentially shift fence-sitting individuals toward acceptance of the supposedly protective military aggression, precisely the attitudinal shift that allowed Bush administration's 2003 invasion of Iraq (76, 77).

The Costs of War Project has since documented that direct violence in Iraq, Afghanistan and the broader post-9/11 theatres has killed at least 900,000 people (78). *How Death Outlives War* notes that Iraqi physicians have observed higher prevalence of diverse birth defects, including cardiac malformations, and childhood cancers such as leukaemia in hard-hit cities like Fallujah, as well as rising cancer mortality reported by health authorities in Basra (79). The report further details how many U.S. veterans continue to suffer sequelae such as PTSD and toxin-related illnesses, though its primary concern is the much larger civilian health burden created by the post-9/11 wars (79). That such carnage nonetheless co-exists with record NFL viewership testifies to the capacity of professional sports to aestheticise war and convert complex geopolitics and loss of human life into consumable narratives of valorising one's own troops and halftime salutes.

### U.S imperialism and the Israeli military assault on Gaza

The Gaza Strip now functions as a sort of necropolitical laboratory, a sporting battleground in a genocidal landscape, with its population rendered hostage to American-Israeli ordnance, spare-part supply chains, and diplomatic vetoes (80). Necropolitics can refer to a sort of system of governance built on the accumulation of power that can dictate death, injury, and debility for populations (81). As of June 2025, Israeli forces have killed a total of 785 Palestinian athletes and sports officials, and destroyed 288 sports facilities, the vast majority in Gaza (82). The enclave's nearly 2 million population, or whatever is left of it, is held captive not only by Israeli artillery but also by the U.S. supply chains and vetoes that sustain violence against Palestinians and keep those weapons flowing. Edward Said, writing in 1977, located Israeli stories of creation and U.S. imperialism on the same ideological axis: both mobilise settler myths of empty land and divine mandate to justify Indigenous erasure (83). Washington arms Israel not because of domestic lobby pressure alone, but because Israel performs imperial policing crucial to U.S. hegemony in the Eastern Mediterranean (84, 85). Israel functions as an unsinkable aircraft-carrier, and its capacity to police the region is rewarded with unconditional political, military and media cover. Sport helps broadcast and amplify this alliance. While Israeli bombs reduced Gaza City's Yarmouk Stadium to a prison camp, ESPN, the NCAA's “March Madness,” and NBA arenas lit up in blue-and-white graphics urging viewers to “stand with Israel,” a public-relations display that echoed State-Department talking points and drowned out calls for a cease-fire in Palestine (84, 86).

These sorts of alignments between competitive sports and media can sit within a broader pattern in which international sports can operate as a sort of mechanism for sportswashing, i.e., the production of normalcy and legitimacy through inclusion of country-sponsored athletes, athletic teams, and nationalist celebration even amid grave human rights violations perpetrated by a given country. In the case of Israel, the routine display of state symbols in sporting venues and the largely unremarked integration and inclusion of athletes who have served in state security institutions can help present militarised nationalism as a sort of culturally ordinary thing. This can help shift attention away from the lived health consequences for Palestinians living under occupation, blockade, and mass violence being perpetrated by an apartheid regime.

Health data leave no room for euphemism. Estimates of tens of thousands of direct deaths and hundreds of thousands of indirect deaths, including the widespread destruction of civilian infrastructure in Gaza have taken hold (87–91). Among the dead and maimed are scores of athletes: the Israeli military have repeatedly shot, jailed, or killed football players, stripped clubs of their pitches, and turned stadiums into detention sites, tactics detailed by sport scholar Jon Dart as an intentional effort to “curtail the emergence” of a Palestinian football (85). Such destruction is inseparable from U.S. logistics: by September 2024 Washington had already committed at least US $22.76 billion to Israel's war effort, including US $17.9 billion in direct security assistance, even as American professional leagues and franchises turned humanitarian language into branding opportunities instead of challenging the violence their own government bankrolls (92). When Palestinian athletes demand a cease-fire, they are therefore not the first to be necessarily inserting “politics” into sport, they are calling out the geopolitical circuitry that already runs through every scoreboard and sponsorship deal.

## Conclusion

The foregoing analyses dislodge a lot of residual confidence that organised sport is a simple public-health good. By tracing the athletic spectacle from the medical clinics to the desert construction sites and then to the smoking ruins of Gaza, we have shown how sport can be situated within the same political economies that govern labor markets, international finance, and armed forces. The dominant sport-health ideology can be damaging to individuals and communities alike and depends on categorical slippage: it could equate brisk walking with repetitive head trauma, voluntary recreation with compulsory wage labour, and personal fitness with corporate marketing. Once these conflations are prised apart, a different landscape could come into view. Elite leagues and mega events do not merely coexist with injury and exploitation, they may be animated by them. The profitable circulation of logos and broadcast rights often presupposes a tier of workers whose heatstroke, fractures, and uncompensated deaths are written off as collateral. Meanwhile, the ceremonial intimacy between professional franchises and the U.S. security apparatus could render foreign military campaigns an extension of the weekend pastime. Bodies harmed by errant bombs in the Middle East may thus occupy the blind spot of health promotion posters that trumpet sport's capacity to bring people together.

Repositioning sport inside the bioethical field could therefore require more than the familiar calls for concussion protocols or supplement regulation. It may demand that we treat labour rights, sponsor accountability, and geopolitical alignment as first-order determinants of population health. The moral and clinical stakes are clear. When a governing body accepts petro-dollars or weapons manufacturers as partners, it may be participating in the upstream production of heart disease and congenital malformation documented among populations living beside oil flares or beneath ordnance trajectories. When a university athletic department withholds wages from its players, it may actually consign many of them to the unstable insurance status now recognised as an independent predictor of shortened life expectancy. When a league affixes camouflage insignia to every jersey and sells field-side advertising to defence contractors, it is not engaging in an entirely neutral tribute, but rather it is risking underwriting the public tolerance necessary for distant battlefields whose epidemiology of burns, amputations, and contaminated aquifers is already catalogued by physicians.

If these entanglements are constitutive rather than accidental, what possibilities remain for turning sport toward health rather than harm? Three avenues present themselves. The first is to dispel the sport-health ideology, as it risks conflating sport with physical activity, obscures widespread injury and long-term health consequences, masks exploitative labour practices, distracts from social and structural determinants of health, reinforces racial hierarchies, and promotes problematic military narratives. Second, the analytic tools of occupational and environmental medicine could be normalised inside sport governance. Heat-stress indices, cumulative-load surveillance, and transparent fatality registries have saved countless lives in mining and construction, and they could do the same for athletes, stadium crews, and migrant cleaners if adopted without exception clauses that are far-reaching for certain sorts of special events or cases. Finally, the regulatory horizon could widen to treat sponsorship as a modifiable risk factor. Just as trans-fat bans or plain-pack cigarette laws severed the link between harmful products and everyday consumption, sport administrators can refuse partnerships that contradict public-health objectives or that legitimate violence abroad. Those who would claim that such steps politicise sport may need to reckon with the empirical record presented here: sport is already organised through distinctly political infrastructures of extraction and projection.

In clinical medicine, a core precept holds that the duty of care extends beyond the presenting symptom to the material conditions that generate illness. If a child's asthma is triggered by mold in a substandard apartment, prescribing inhalers without addressing housing policy might be a bit negligent. The same logic could apply to sport. Yet mainstream bioethics, still magnetised to the bedside and its favoured trolley-car puzzles, rarely follows that principle to its logical endpoint. Its clinical fixation narrows the field's moral lens, pushing questions about wage theft in stadium construction or unpaid collegiate labour to the margins as being domains of policy rather than bioethics. This gatekeeping means that analysts must often first justify why labour exploitation, environmental contamination, or militarised branding even count as ethical concerns - a pre-screening that protects the very structures producing harm. By cordoning off structural determinants as someone else's problem, bioethics risks becoming the intellectual handmaiden of the industries it ought to interrogate. A sport bioethics worth the name may need to therefore break free of the clinic's gravitational pull and treat extraction, inequity, and imperial spectacle as central, not peripheral, to its mandate.

It is possible that sport social workers might be in a better position than sports medicine practitioners to realize the importance of prioritizing health over performance (93). If they are attuned to some of the structural dynamics at play, they could possibly speak to the labor exploitation element (and possibly connect athletes to unions). For social workers that are more focused on mental health, it might be important to create a space for them to discuss (with both current and former athletes) how the very structure of sport (competition, hierarchy, and constant evaluation) has impacted their psychological health, interpersonal relations, and their relationships to their body (e.g., trained to ignore pain signals; the body belongs to the coach, not themselves, etc.). Focusing on this structural element of sports can provide analytic clarity for both the social worker and athlete since often the blame is placed on a supposed bad apple (e.g., bad coach).

To commend exercise while ignoring the heat index that killed Nepalese roofers at Lusail, or to celebrate team spirit while the halftime show normalises the bombardment of Rafah, is arguably a form of scholarly and professional negligence. A bioethics of sport adequate to the twenty-first century could recognize that the pitch is contiguous with the factory floor and the battlefield, and that health cannot be advanced in one domain while it is sacrificed in another. Our task is therefore to dismantle the conditions under which physical activity thrives on debility and to imagine cultures where the exhilaration of collective play no longer depends upon the unseen labor, broken bodies, and silent funerals of others.
